# Uncovering the reasons behind the failure of pastoralists in adopting climate change adaptation strategies

**DOI:** 10.1038/s41598-024-70818-4

**Published:** 2024-09-04

**Authors:** Mohsen Sharaftmandrad, Ahmad Abedi Sarvestani, Mohammadreza Shahraki, Mohammad Hassanzadeh Nafooti

**Affiliations:** 1 Department of Ecological Engineering, Faculty of Natural Resources, University of Jiroft, 8th Km of Jiroft - Bandar Abbas Road, Jiroft, Iran; 2 Agricultural Extension and Education Department, Faculty of Agricultural Management,, Gorgan University of Agricultural Sciences and Natural Resources, Gorgan, Iran; 3 Researcher of Rural Development and Social Issues in the Field of Natural Resources and Agriculture, Gorgan, Iran; 4 Department of Natural Resources, Meybod Branch, Islamic Azad University, Meybod, Iran

**Keywords:** Drought, Livelihood, Livestock, Rangeland, Structural equation modeling, Climate-change adaptation, Climate-change policy

## Abstract

Climate change has caused pastoralists to face serious challenges all around the world. To reduce climate change vulnerability, adaptation strategies need to be adopted by pastoralists. In this regard, the present research was done to seek the reasons for the failure of the northeastern pastoralists of Iran in adopting climate change adaptation strategies. The study is descriptive, which conducted by a field survey. The target population included 249 pastoralists from 7 pastoral units, of whom 148 people were selected as sample size using the stratified random sampling technique. The survey instrument was a researcher-made questionnaire. The content validity and face validity of the questionnaire were checked by the experts. Convergent validity was also confirmed based on the average variance extracted (AVE). Cronbach’s α coefficient and composite reliability (CR) were used to evaluate the internal consistency of the questionnaire. The results showed that social and, regulatory and insurance components were the most critical internal and external weaknesses of the pastoralists’ failure in adopting climate change adaptation strategies, respectively. Structural equation modeling showed that external weaknesses had positive and significant effects on internal weaknesses of the pastoralists’ failure in adopting climate change adaptation strategies.

## Introduction

Trend analysis of long-term meteorological data and weather forecasting show apparent changes in the global climate. Climate change is becoming one of the most severe threats that humanity is facing^1^. The rise in temperature, precipitation pattern changes^2,3^, and their consequences (extreme weather events such as flash floods, forest fires, heat waves, and droughts) adversely affect human life and livelihood all over the world^4,5^. Therefore, the proper responses to climate change are critical^6^. One of the responses is the adaptation to climate change^7^, which has now become one of the main priorities of different societies^8^. By adapting to climate change, the resulting vulnerability and risks can be reduced^9^, otherwise, climate change can have negative impacts on society^10^.

There are interactions between climate and human^8^. Knowing these interactions will help us understand the risks caused by climate change to take necessary actions to climate change^11^. In addition to understanding the causes and consequences of climate change, it is necessary to take appropriate actions to deal with the adverse effects of climate change. This is a motivating factor to take the preventive measures of climate change^12^. Therefore, understanding the climate change and its impacts on local ecology is an important starting point in dealing with its adverse effects^13^.

Adaptation to climate change refers to the response to the actual climatic stimuli or the prediction of climate change and its adverse effects, providing the basis for using the beneficial opportunities^14^. Adaptation can include small-scale, short-term actions or using opportunities arising from climate change to reduce damages^15^. Adaptation refers to the appropriate actions taken to adjust to new climatic conditions and taking advantage of the resulting changes^16^. For this reason, adaptation can be considered as the act of adjusting economic, social, and environmental conditions to make them suitable for the consequences of climate change^1^. It does not only focus on modifying the damages caused by climate change, but also emphasizes on the use of beneficial opportunities. To empower local communities, it is necessary to know the prerequisites and strategies of climate change adaptation^17,18^. On a small scale, livelihood adaptation to climate change refers to the act of adjusting household livelihood, resources, and activities in order to increase its ability to survive against climate change^19^. This issue is more severe for many rural households, because they are directly affected by climate change due to their weak economy and dependence on natural resources^20^.

Due to the solid financial dependence on rangelands, pastoralists are considered as one of the low-income social classes. Climatic change and its resulting risks, especially drought, have adverse effects on the rangelands condition and livestock^21–23^. The reduction of vegetation, forage, and water sources, the exacerbation of livestock diseases, and the distortion of production systems have negative effects on pastoralism, weakening their adaptive capacity and exacerbating food insecurity^21^. To avoid the consequences of climate change, many pastoralists have no choice but to adopt adaptation strategies^24^. They take different actions to combat the consequences of climate change and to maintain their livelihood e.g., earning a living without animal husbandry, migrating, and diversifying income sources^25,26^. These actions refers to a set of asset utilization activities and methods that people practice to achieve livelihood goals^26,27^.

There are different livelihood adaptation strategies under crisis conditions^28^. Adopting climate change adaptation strategies is not enough^29,30^ and resource mobilization, decision-making, planning, and implementation of proper policies by government institutions and local authorities are required^31,32^. In other words, it is necessary to pay attention to government structures and various social, economic, political, educational, etc. elements to receive a positive response and succeed in adapting to climate change^33–35^.

Governments need to implement local-scale measures to empower local economies in the face of adverse impacts of climate change^21,36^. In addition to adapting to climate change, local communities need to look at appropriate measures to minimize risk exposure. They should take proper prevention and mitigation measures to reduce the climate change risks. Development, adoption, and implementation of new policies, proper management of natural resources, development of infrastructures, and improvement of the locals’ participation are among the factors affecting the local communities’ adaptation to climate change^34,37^. The incomplete application of preventive principles against climate change risks challenges the success of local communities’ adaptation to climate change due to their lack of awareness^3,38^. Local communities’ adaptation to climate change is affected by the lack of human and financial resources, the lack of experience and expertise, and the lack of government support^39^. Lack of active labor, lack of income opportunities, lack of access to sufficient credits and facilities, and lack of access to markets for product sale are the main determinant factors of local communities’ adaptation to climate change^40–43^. Local communities have inadequate response to climate change adaptation strategies due to lack of accurate planning, misperception of time concerning climate change^3,44^, avoiding existing adaptation strategies, and factors limiting the use of local knowledge^45,46^.

Financial resources and new technologies should be provided as a complement to indigenous knowledge to achieve the goals of climate change adaptation and to reduce the vulnerability of local communities to climate change^47^. Early warning systems, infrastructure facilities, emergency preparedness, and good income status play an essential role on human adaptation to climate change^48,49^. The lack of environmental knowledge and education, the lack of adaptation incentives, the gaps in the law, and the lack of government’s support have doubled the concerns related to climate change adaptation. Access to climate information, credit, and extension services, rearing local breeds of livestock adapted to the region conditions, and storing fodder are among the factors that have a direct impact on the adaptive capacity of pastoralists to climate change^50^. Lack of water and forage in rangelands is one of the most important challenges for pastoralists’ adaptation to climate change^51^. Timely response to climate change, adequate flexibility, matching the type of livestock with weather conditions and vegetation, management of pests and plant diseases, moving the accommodation, and livestock management in rangelands have increased the pastoralists’ adaptive capacity to climate change^52^.

On the other hand, various studies have pointed out the influence of personal characteristics of pastoralists on their response to climate change such as age^21,50^, household size^21,53,54^, literacy^22,41,53,54^, access to climatic information^21,50,55,56^, herd size^21,53,57,58^, having agricultural land^57^, animal husbandry experience^15^, the amount of income from animal husbandry^15,41,55^, the amount of income from non-animal husbandry^21,53^, having equipment and facilities^54^, and participating in training courses and meetings^15,54^.

Iranian pastoralists are also affected by climatic change and its effects on rangelands. A decrease in the precipitation, changes in its patterns, and increased temperature had accelerated the rangeland degradation, intensified the lack of forage and drinking water for livestock, and increased the spread of animal and human diseases in the northeastern rangelands of Iran. It can be seen that the region annual rainfall in 2023 has reduced more than 40%, limiting drinking water for livestock and pastoralists. These conditions harmed the pastoralism system and the livelihood of Turkmen pastoralists in the area, facing them with significant challenges. Consequently, these conditions encouraged pastoralists (more than 60%) to leave animal husbandry in the region and to migrate temporarily. In addition to the challenges and problems related to climate change, some pastoralists quit animal husbandry due to the non-profitable nature of pastoralism, the lack of rangeland forage, and the increase in animal husbandry costs. Although Turkmen pastoralists have a long history in animal husbandry, they have rich indigenous knowledge, in particular, knowledge of adaptation to climate change, they failed in adopting climate change adaptation strategies. Since, pastoralists’ adaptation to climate change is crucial to create a sustainable livelihood system and ensure food security in rangeland ecosystems, this study aimed to (1) uncover internal and external weaknesses of pastoralists in adopting climate change adaptation strategies, (2) investigate the relationship between pastoralists weaknesses in adopting climate change adaptation strategies and their personal characteristics, and (3) assess the effect of external weaknesses on internal weaknesses of pastoralists in adopting climate change adaptation strategies.

## Materials and methods

### Study area

**Fig. 1:**
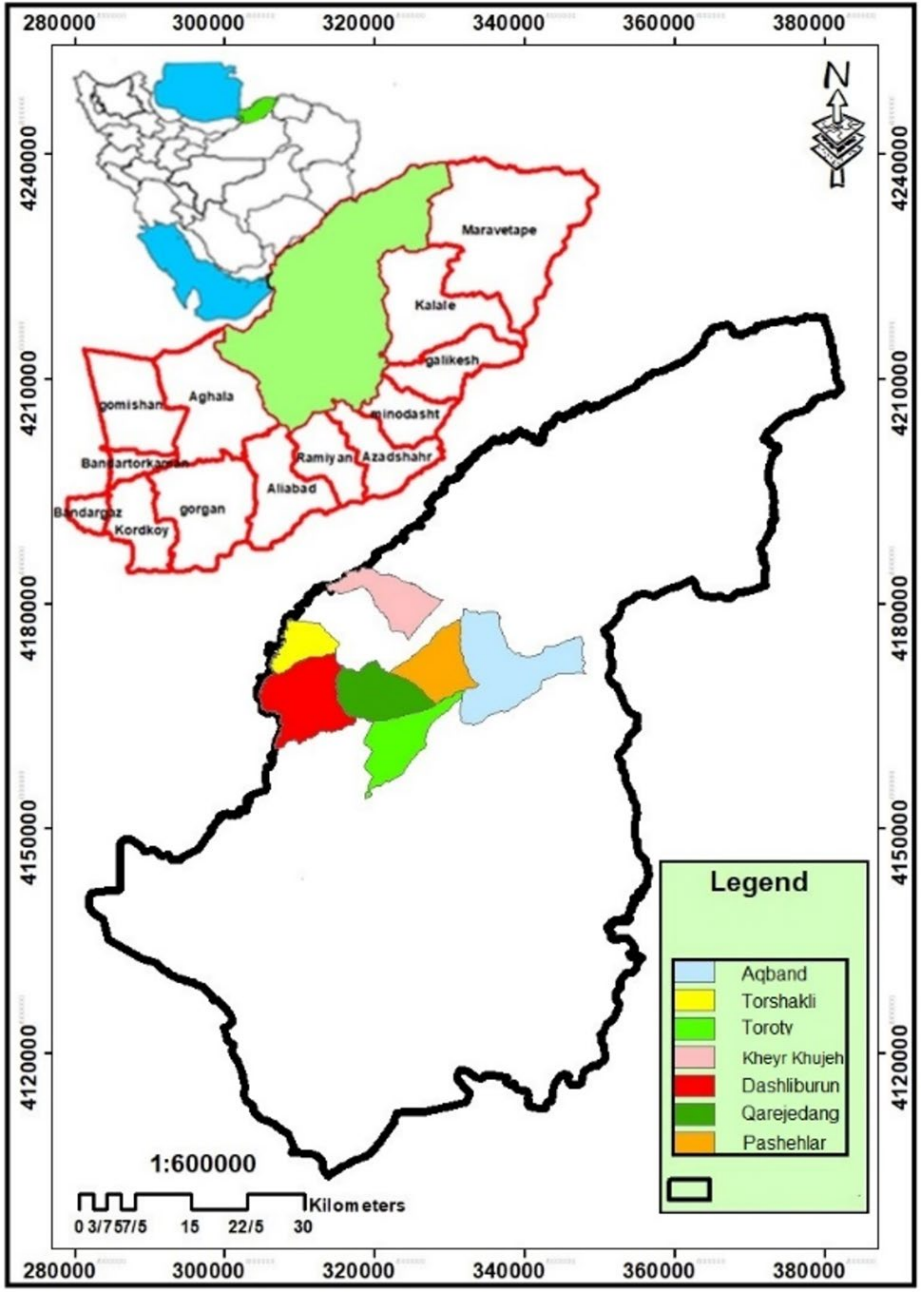
The map of the study area and its seven pastoral units (Aqband, Dashliburun, Qarejedang, Pashehlar, Toroty, Torshakli, and Kheyr Khujeh). *Source* Mapped by the authors using ArcGIS Desktop V. 10.8.

Qarah Dang rangelands were selected as study area (37°36ʹ23ʺN-54°49ʹ41ʺE to 37°44ʹ36ʺN-55°11ʹ39ʺE). These rangelands are a part of the Atrak river basin in Golestan province, Iran (Fig. 1). Qarah Dang rangelands cover an area about 228.9 km^2^. The area is relatively flat. The average annual rainfall is 249.6 mm. The area climate is semi-arid according to the modified De Martonne classification, indicating the low potential for providing water resources. The average annual potential evaporation is estimated to be 3 times greater than the annual rainfall. The main river of the region run dry for most of the year and river bed was plowed and converted into rainfed croplands by some pastoralists.

There are 116 plant species from 32 families in the study area. Poaceae and Asteraceae are the most abundant families. Climatic limitations (low precipitation, its uneven distribution, and drought), an extending grazing season in the rangeland, and rangeland to cropland conversion have severely affected the vegetation, so that most of the perennial palatable species have disappeared and invasive and poisonous species dominated. In some parts, soil is left bare where vegetation cover is absent or almost absent. Therefore, a special situation has arisen in the region, increasing the proportion of annual species in vegetation composition.

All the pastoralists in the region are Turkmen, occupied by animal husbandry and agriculture. The rangelands are annually grazed by herds from the end of November to the end of March (4 months based on their grazing permit). However, livestock grazing may be extended to the end of April due to the lack of rangeland forage.

### Data collection

This study is a survey research, quantitative in terms of the nature and applied in terms of the objective. The statistical population includes 249 Turkmen pastoralists belonging to seven pastoral units, of which 148 pastoralists were selected as the sample size based on Krejcie and Morgan Table (Table 1). The pastoralists were selected using stratified sampling scheme. So, samples were divided among the seven pastoral units. The number of samples was allocated based on the population of each pastoral unit.

**Table 1:** The number of samples allocated to different pastoral units.

Pastoral unit	Population	Sample size
Aqband	68	40
Dashliburun	46	27
Qarejedang	32	19
Pashehlar	32	19
Toroty	38	23
Torshakli	15	9
Kheyr Khujeh	50	20
Total	249	148

The research tool was a researcher-made questionnaire. The initial items were listed based on the research objectives and literature review. To modify and localize the initial items, 10 individual interviews were conducted with the organizational experts (General Department of Natural Resources and Watershed Management of Golestan Province) and experienced pastoralists. The interviewees were selected by snowball method. In this stage, 82 items were listed. After removing duplicate and irrelevant items, 49 items were finalized.

The content validity and face validity of the questionnaire were checked by the experts. Convergent validity was also confirmed based on the average variance extracted (AVE). Cornbrash’s α coefficient and combined reliability (CR) were calculated to evaluate the internal consistency of the questionnaire (Table 2).

**Table 2:** The Cronbach’s alpha coefficient of the components.

Component	Number of items	Cornbrash’s alpha	Composite reliability (CR)	Average variance extracted (AVE)
Internal weaknesses	18	0.795	0.869	0.570
Economic	3	0.758	0.845	0.655
Knowledge-based	3	0.758	0.790	0.576
Communicational	2	0.849	0.930	0.869
Ecological	3	0.875	0.923	0.800
Social	4	0.902	0.932	0.774
Psychological	3	0.808	0.886	0.723
External weaknesses	16	0.857	0.900	0.693
Infrastructural	5	0.798	0.861	0.561
Supportive	5	0.875	0.909	0.668
Regulatory and insurance	2	0.821	0.862	0.758
Market	4	0.884	0.920	0.742

The first part of the questionnaire was related to the personal characteristics of the respondents, which was investigated using 16 items (Table 3), of which nine and seven items were respectively related to personal and economic characteristics of the respondents.

**Table 3:** Personal characteristics of the pastoralists in the region.

Characteristic	Class	Frequency	Frequency percentage
Age (year)	< 40	36	24.3
	40–50	50	33.8
	50 <	62	41.9
	Mean = 50.28	Min = 26	Max = 82
Marital status	Married	131	88.5
	Single	17	11.5
Literacy level	Illiterate	62	41.9
	Elementary	57	38.5
	Middle school,	4	2.7
	High school and higher	25	16.9
Family size	< 4	16	10.8
	4	45	30.4
	5	50	33.8
	6	20	13.5
	7 ≤	17	11.5
	Mean = 5.01	Min = 2	Max = 12
Number of family members involved in animal husbandry	1	9	6.1
	2	59	39.9
	3	66	44.6
	Mean = 2.20	Min = 0	Max = 3
Number of livestock	< 100	21	14.2
	100–200	67	45.3
	200–300	40	27.0
	300 <	20	13.5
	Mean = 200.14	Min = 70	Max = 480
Years of experience in animal husbandry	1–10	29	19.6
	10–20	18	12.2
	20–30	46	31.1
	30 <	55	37.2
	Mean = 28.41	Min = 4	Max = 55

The second part of the questionnaire was related to internal and external weaknesses of the failure of pastoralists in adopting climate change adaptation strategies. Internal weaknesses included 6 components (economic, knowledge-based, communication, ecological, social, and psychological components), which were evaluated through 18 items (Table 4). External weaknesses included infrastructural (5 items), supportive (5 items), regulatory and insurance (2 items), and market (4 items) components. In total, external weaknesses were evaluated through 16 items (Table 5).

**Table 4:** Comparison of the internal weaknesses of the pastoralists’ adaptation to climate change.

Component	Item	Min	Max	Mean	SD	Item rank	Overall rank
Economic	Inadequate financial resources and inability to invest (limited access to financial resources)	1	5	2.878	0.910	2	16
	Inability to receive loans and bank facilities to compensate for damages	1	5	2.872	0.942	3	17
	Increasing animal husbandry costs and disproportion between costs and incomes	1	5	3.243	0.854	1	11
Knowledge-based	Low level of knowledge and awareness	1	5	3.615	0.752	1	1
	Low level of literacy to implement climate change adaptation strategies	1	5	3.514	0.845	2	6
	Lack of proper training programs and conducting targeted training courses	1	5	2.601	0.855	3	18
Communication	Low access to experts and extension specialists	1	5	3.405	1.022	2	10
	Low access to media and virtual communication channels	1	5	3.473	0.845	1	8
Ecological	Crisis in quantity and quality of drinking water for livestock and family	1	5	3.547	0.860	2	4
	Decrease in the rangeland forage quality and diversity	1	5	3.568	0.934	1	3
	Mismatch between the rangeland capacity and stocking rate	1	5	3.250	0.968	3	12
Social	Increasing conflict due to communal exploitation	1	5	3.588	0.918	1	2
	Poor participation in restoration practices	1	5	3.541	0.985	2	5
	Low level of trust among themselves and with experts	1	5	3.480	0.929	3	7
	Failure to pay attention to the recommendations of experts and extension specialists	1	5	3.412	0.872	4	9
Psychological	Lack of risk-taking to implement climate change adaptation strategies	1	5	3.182	0.783	1	13
	Loss of hope for the future	1	5	3.142	0.765	2	14
	Decreased motivation and devotion in pastoral life	1	5	2.932	0.967	3	15

Range of answers: (1: very little; 2: little; 3: to some extent; 4: a lot; 5: very much.

**Table 5:** Comparison of the internal weaknesses of the pastoralists’ adaptation to climate change.

Component	Item	Min	Max	Mean	SD	Item rank	Overall rank
Infrastructural	Lack of infrastructure for storing drinking water such as pools and reservoirs	1	5	3.047	0.957	3	14
	Lack of crisis warning systems	1	5	3.041	0.880	4	15
	Poor health infrastructure	1	5	2.953	0.906	5	16
	Lack of planning for rainwater harvesting	1	5	3.318	0.881	1	11
	Legal restrictions on the reconstruction and strengthening of houses and barns	1	5	3.257	0.927	2	13
Supportive	Non-payment of low-interest loans by the government	1	5	3.291	0.935	5	12
	Improper provision of subsidized fodder government services in times of crisis	1	5	3.439	0.818	2	8
	Non-allocation and inappropriate use of government credits for strategies related to climate change	1	5	3.405	0.872	3	9
	Lack of conditions to create complementary jobs in the region	1	5	3.466	0.852	1	6
	Poor veterinary services	1	5	3.399	0.909	4	10
Regulatory and insurance	Lack of transparency of insurance services for livestock and rangeland and not covering damages when claiming on the insurance	2	5	3.642	0.780	1	2
	Lack of specialized measuring and monitoring of climate change	2	5	3.446	0.785	2	7
Market	Uneconomical herding due to extreme price fluctuations	2	5	3.662	0.743	1	1
	The price of livestock is not proportional to the supplementary feed in the market	2	5	3.514	0.829	3	4
	Lack of easy access to local and regional markets to supply livestock products	2	5	3.493	0.778	4	5
	Inflation and market fluctuations in buying inputs and selling livestock products	1	5	3.541	0.891	2	3

Range of answers: (1: very little; 2: little; 3: to some extent; 4: a lot; 5: very much.

### Data analyses

Descriptive and inferential statistics were done in IBM SPSS Statistics 25.0. In the descriptive statistics, frequency, frequency percentage, mean, minimum and maximum of the respondents’ personal characteristics were computed. The interval of standard deviation from the mean (ISDM) was calculated to classify the effect of internal and external weaknesses on the failure of pastoralists in adopting climate change adaptation strategies. Based on the sum of the values, components were classified into four classes:

$$\begin{gathered} {\text{A}}:{\text{ A }} < {\text{ Mean}} - {\text{St}}.{\text{d}} \hfill \\ {\text{B}}:{\text{ Mean}} - {\text{St}}.{\text{d }} \le {\text{ B }} < {\text{Mean}} \hfill \\ {\text{C}}:{\text{ Mean }} \le {\text{ C }} < {\text{ Mean}} + {\text{St}}.{\text{d}} \hfill \\ {\text{D}}:{\text{ Mean}} + {\text{St}}.{\text{d }} \le {\text{ D}} \hfill \\ \end{gathered}$$

Spearman’s correlation coefficient was used to investigate the relationships between the internal and external weaknesses of the failure of pastoralists in adopting climate change adaptation strategies and their personal characteristics. Mann–Whitney test was used to compare pastoralists in terms of response to climate change adaption strategies in dichotomous groups. In order to compare the internal and external weaknesses of the failure of pastoralists in adopting climate change adaptation strategies, the total score of each dimension was divided by its number of items to calculate the unweighted linear combination. Structural equation modeling was used to assess the effect of external weaknesses on internal weaknesses of the failure of pastoralists in adopting climate change adaptation strategies. Hypothetical patterns of direct and indirect relationships between a set of observed and latent variables are investigated in structural equation modeling. Latent variables are the main factors that are displayed in a model or conceptual model. Observed variables are items or questions related to measuring the main factors. This method is a special causal structure between a set of latent variables and observable variables. Using the structural equation modeling, the relationships between the latent variables and the relationships between measurement items of each latent variable and the related variable were investigated. The path coefficient was used to show the e causal relationship between the latent and observable variables. The strength of the relationship between the latent and observable variables is represented by the factor loading. Factor loading ranges between 0 and 1. If the factor loading is less than 0.3, the relationship is considered weak and it is ignored. A factor loading between 0.3 and 0.6 is acceptable, and if it is greater than 0.6, it is very desirable. SmartPLS 3 software was used to perform the calculations of this method.

### Ethics approval and consent to participate

All experimental protocols were approved by Review Board of Department of Ecological Engineering, Faculty of Natural Resources, University of Jiroft, Iran. All methods were carried out in accordance with relevant guidelines and regulations. Informed consent was obtained from all participants.

## Results

### Personal characteristics of pastoralists

The main personal characteristics of the respondents are summarized in Table 3. The average age of the respondents was 50.2 years, ranging between 26 and 82 year. 88.5 percent of the respondents were married and the rest were single. The mean family size was 5, ranging between 2 and12. 44.6 percent of pastoralists had involved 3 family members in animal husbandry (the highest frequency). Also, 41.9 percent of the respondents were illiterate who did not have the ability to read and write. The pastoralists had averagely 200 heads of livestock, ranging between 70 and 480 heads of livestock. Meanwhile, most of the pastoralists (45.3%) had 100–200 heads of livestock, which are considered as small pastoralists. 50.6 and 12.7 percent of the pastoralists )the highest and the lowest frequencies( had less than 40 years and more than 50 years of experience in animal husbandry, respectively. The minimum, maximum and mean experience of animal husbandry were 26, 57 and 39 years, respectively.

### Internal and external weaknesses of the failure of pastoralists in adopting climate change adaptation strategies

Six items including “increasing animal husbandry costs and disproportion between costs and incomes”, “low level of knowledge and awareness”, “low access to media and virtual communication channels”, “decrease in the rangeland forage quality and diversity”, “increasing conflict due to communal exploitation” and “lack of risk-taking to implement climate change adaptation strategies” with the highest average values were the first item of each component for the failure of pastoralists in adopting climate change adaptation strategies. Based on internal items comparison, “low level of knowledge and awareness” from the *knowledge-based* component with an average of 3.615, “increasing conflict due to communal exploitation” from the *social* component with an average of 3.588 and “decrease in the rangeland forage quality and diversity” from the *ecological* component with an average of 3.568 were the first three limitations of the pastoralists’ adaptation to climate change (Table 4).

Concerning the external weaknesses of the pastoralists’ adaptation to climate change, “lack of planning for rainwater harvesting” from the *infrastructure* component, “lack of conditions to create complementary jobs in the region” from the *supportive* component, “lack of transparency of insurance services for livestock and rangeland and not covering damages when claiming on the insurance” from the *regulatory and insurance* component and “uneconomical herding due to extreme price fluctuations” from the *market* component with the highest mean values were the most important external weaknesses of the failure of pastoralists in adopting climate change adaptation strategies. Also, the three items “uneconomical herding due to extreme price fluctuations” and “inflation and market fluctuations in buying inputs and selling livestock products” from the market component and “lack of transparency of insurance services for livestock and rangeland and not covering damages when claiming on the insurance” from the *regulatory and insurance* component were the most important external weaknesses of the failure of pastoralists in adopting climate change adaptation strategies (Table 5).

The comparison of internal and external components showed that the pastoralists are faced to internal (social (3.505) and ecological (3.455)) and external (market (3.552) and regulatory and insurance (3.544)) weaknesses in adopting climate change adaptation strategies. *Psychological* and *economic* components from internal weaknesses and *supporting* and *infrastructural* components from external weaknesses had the least importance for the pastoralists’ adaptation to climate change (Table 6).

**Table 6:** Comparison of internal and external weaknesses of the failure of pastoralists in adopting climate change adaptation strategies.

Dimension	Component	Min	Max	Rank	Mean	SD	Unweighted linear combination
Internal	Economic	3	15	6	8.993	2.218	2.998
	Knowledge-based	5	13	4	9.730	1.721	3.243
	Communication	2	10	3	6.787	1.741	3.439
	Ecological	4	15	2	10.365	2.472	3.455
	Social	4	20	1	14.020	3.263	3.505
	Psychological	3	15	5	9.257	2.135	3.086
External	Infrastructural	6	25	4	15.615	3.374	3.123
	Supportive	6	25	3	17.000	3.582	3.400
	Regulatory and insurance	4	10	2	7.088	1.380	3.544
	Market	8	20	1	14.209	2.788	3.552

Meanwhile, 39.9% and 40.5% of the respondents had high internal and external weaknesses in adopting climate change adaptation strategies, respectively (Table 7).

**Table 7:** The extent of internal and external weaknesses for the failure of pastoralists in adopting climate change adaptation strategies.

Dimension	Class	Mean	SD	Min	Max	Frequency	Frequency%
Internal	Low (31–49.65)	59.24	9.59	31	84	20	13.5
	Medium (49.66–59.24)					45	30.4
	High (59.25–68.83)					59	39.9
	Very high (68.84–84)					24	16.2
External	Low (25–44.54)	53.91	9.37	25	80	27	18.2
	Medium (44.55–53.91)					40	27.0
	High (53.92–63.28)					60	40.5
	Very high (63.29–80)					21	14.2

### The relationship between personal characteristics of pastoralists and their response to climate change adaption strategies

The number of livestock had a positive and significant relationship with the pastoralists’ response to climate change adaption strategies. The more the number of livestock, the more their weaknesses in response to climate change adaption strategies. The number of family members involved in animal husbandry, years of experience in animal husbandry, the amount of income from animal husbandry, the amount of income from non-animal husbandry jobs, the level of understanding of climate change, the amount of experience in facing climate change in the rangeland, age, literacy level, and the level of knowledge and awareness of climate change had negative and significant relationships with the pastoralists response to climate change adaption strategies. In other words, by increasing each of these variables, the pastoralists’ weaknesses in response to climate change adaption strategies had been increased (Table 8).

**Table 8:** Correlation between personal characteristics of pastoralists and their response to climate change adaption strategies.

Variable	Correlation coefficient (r)	Sig	Relationship
Age	− 0.169*	0.035	Negative
Literacy level	− 0.226*	0.015	Negative
Family size	− 0.127	0.094	Non-significant
Number of family members involved in animal husbandry	− 0.317**	0.008	Negative
Number of livestock	0.421**	0.000	Positive
Years of experience in animal husbandry	− 0.388**	0.000	Negative
The amount of animal husbandry income	− 0.278*	0.010	Negative
The amount of non-animal husbandry income	− 0.386**	0.000	Negative
The level of knowledge and awareness of climate change	− 0.219*	0.011	Negative
Understanding of climate change	− 0.338**	0.000	Negative
The amount of experience in facing climate change	− 0.298**	0.000	Negative
The level of environmental awareness in adapting to climate change	− 0.111	0.073	Non-significant
Access to weather information	0.099	0.270	Non-significant

*Significant at the 95%; **Significant at the 99%.

Based on Table 9, pastoralists were different in terms of response to climate change adaption strategies, depending on having jobs other than animal husbandry, participating in educational and extension courses, and having agricultural equipment and tools. In other words, pastoralists who had jobs other than animal husbandry, had participated in educational and extension courses related to climate change, and also had agricultural equipment and tools (especially tractors and water tankers). They had more adaptive capacity to climate change (Table 9).

**Table 9:** Comparison of pastoralists in terms of response to climate change adaption strategies in dichotomous groups using Mann–Whitney U test.

Variable	Class	Mean	U value	Sig
Marital status	Married	73.79	1020.000	0.574
	Single	80.00		
Having jobs other than animal husbandry	Yes	51.94	2523.500	0.001**
	No	97.43		
Having agricultural lands	Yes	77.54	2389.000	0.303
	No	70.16		
Participating in educational and extension courses	Yes	41.40	2217.500	0.000**
	No	108.74		
Using credit services and getting loans	Yes	78.49	2288.500	0.404
	No	72.34		
Having agricultural equipment and tools	Yes	49.77	2051.000	0.000**
	No	99.01		

*Significant at the 95%; **Significant at the 99%.

### Modeling the effect of external weaknesses on internal weaknesses

**Fig. 2:**
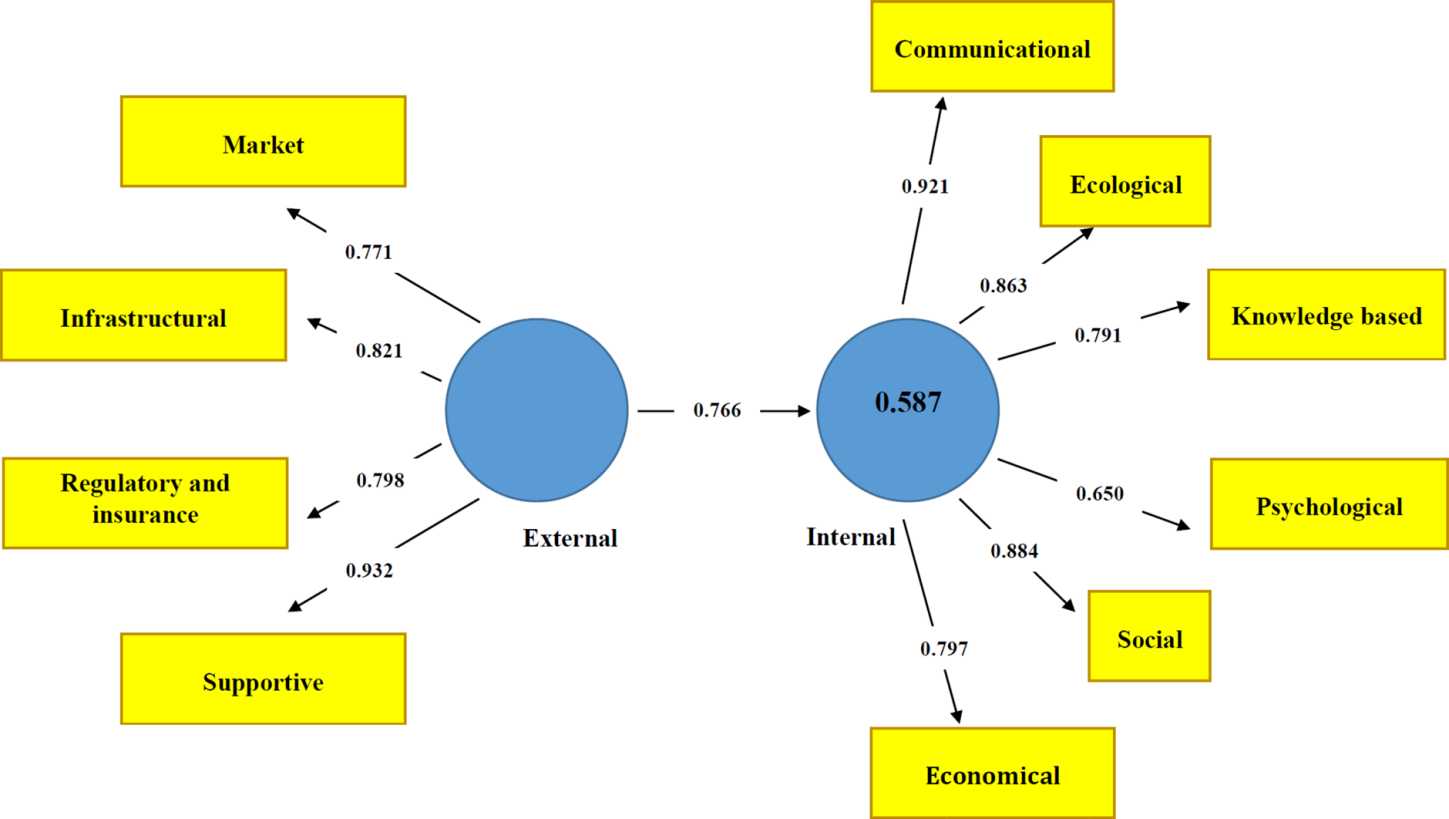
The effect of external weaknesses on internal weaknesses of the failure of pastoralists in adopting climate change adaptation strategies.

The results of the modeling the effect of external weaknesses and internal weaknesses of the failure of pastoralists in adopting climate change adaptation strategies are shown in Table 10. External weaknesses had a positive and significant effect on the internal weaknesses. In this regard, the *supportive* component with a coefficient of 0.932 (external dimension) and the *communication* component with a coefficient of 0.921 (internal dimension) were the most important weaknesses of the failure of pastoralists in adopting climate change adaptation strategies (Fig. 2).

**Table 10:** The effect of external weaknesses on internal weaknesses of the failure of pastoralists in adopting climate change adaptation strategies.

Relation	Path coefficient	t	Sig	Result
External weaknesses → Internal weaknesses	0.766	24.028	0.000	Approved

## Discussion

### Internal weaknesses of the failure of pastoralists in adopting climate change adaptation strategies

Most of the pastoralists had experienced high to moderate internal weaknesses. This finding shows that the failure of pastoralists in adopting climate change adaptation strategies is mainly related to the weaknesses associated with the nomadic life in rangelands. These weaknesses are part of a triangular framework (livestock, pastoralist, and rangeland). In other words, the level of pastoralists’ knowledge, their communication and social interactions, their cooperation and solidarity, livestock, fodder and water management, and also their financial status can play an essential role in reducing the internal weaknesses of their failure in adopting climate change adaptation strategies^39^.

The social component was considered the most critical internal weakness of the failure of pastoralists in adopting climate change adaptation strategies. Shared use of rangelands increased disputes and conflicts between the pastoralists because of the demand of some pastoralists for more shares of rangeland. This finding is consistent with the results of other studies^34,37^. The lack of border restrictions in livestock grazing management has caused pastoralists to compete in the rangeland exploitation even in droughts. So the competition in the rangeland exploitation, land and vegetation degradation, and reduction in the quantity and quality of fodder have doubled pastoralists’ vulnerability to climate change. On the other hand, the conflicts between pastoralists in common use of rangelands resulted in the reduction of intragroup (between pastoralists) and intergroup (between pastoralists and experts) trust, which has weakened their participation in rangeland restoration activities. This made pastoralists not to pay much attention to the recommendations of extension specialists to adopt climate change adaptation strategies. As the conflicts increase and the social trust decreases, a suitable response will not be received in adopting climate change adaptation strategies due to the lack of participation and responsibility in rangeland restoration activities. This led to competition among the pastoralists for more exploitation of the rangeland in critical conditions, resulting in ecological and environmental challenges. The decrease in precipitation, its uneven distribution in the region, changes in the rainfall regimes, and extreme temperature fluctuations have caused drought in the area. Considering the livelihood dependence of the pastoralists on vegetation and fodder in the rangeland, the reduction of rangeland forage has forced them to buy supplementary feed, creating limitations to adapt to climate change. On the other hand, the issues related to quantity and quality of drinking water are other reasons of the failure of pastoralists in adopting climate change adaptation strategies. Such issues can be solved by buying and transporting bulk water volumes in trucks to pastoral units, imposing impose extra costs on the pastoralists.

Communication and knowledge weaknesses were the other factors limiting pastoralists’ response to climate change adaptation strategies. The environmental knowledge and education are of the significant determinants of adaptive capacity of pastoralists to climate change^48,49^. The low literacy levels amongst the pastoralists limit the number of selected adaptation strategies in the face of climate change. So, illiteracy or low literacy level limits the pastoralists’ access to communication channels, especially the information provided on the Internet. Nonfamiliarity with climate change adaptation strategies and not taking advantage of successful experiences offered by the different areas cause problems in the pastoralists’ adaptation to climatic fluctuations. Therefore, planning targeted trainings, conducting face-to-face training courses, and visiting successful regions can be effective in improving the adaptive capacity of pastoralists to climate change.

### External weaknesses of the failure of pastoralists in adopting climate change adaptation strategies

The results showed that more than two-fifths of the pastoralists failed in adopting climate change adaptation strategies due to external weaknesses. In addition to intergroup weaknesses, there were intragroup weaknesses (outside the rangeland) limiting pastoralists’ response to climate change adaptation strategies, such as management, policy-making, and supportive infrastructures. The intragroup weaknesses had a positive effect on the internal weaknesses of the failure of pastoralists in adopting climate change adaptation strategies. The findings of structural equation modeling also confirmed the positive effect of external weaknesses on internal weaknesses.

Market was the most important component of the failure of pastoralists in adopting climate change adaptation strategies. Climate change has reduced forage quantity and quality and water resources in the study area rangelands. These conditions made pastoralists to buy supplementary fodder and drinking water for livestock, increasing the cost of herding in rangelands. Therefore, pastoralists sell part of their livestock in local markets to meet the family’s livelihood expenses. As a result, the herds become progressively smaller, which is not economical for the pastoralists. The high cost-to-income ratio, intermediation, and price volatility of live livestock in the markets have limited the adaptive capacity of pastoralists to climate change. On the other hand, the sharp increase of fodder price in the market and its disproportion with live-livestock price have reduced income and saving performance of pastoralists. Therefore, most pastoralists are forced to buy fodder for a high price, doubling their adaptation problems. Owning agricultural land plays an important role in the adaptation of pastoralists to climate change^42,57,59^. The high inflation rate, input prices fluctuation, and the lack of easy access to local and regional markets have reduced pastoralists’ ability to succeed in adopting climate change adaptation strategies. Therefore, access to markets for the sale of animal products plays an important role in facilitating the adaptation process of pastoralists in the face of climate change^40–43^.

As the results showed, regulatory and insurance component was one of the important factors of the failure of pastoralists in adopting climate change adaptation strategies. The lack of transparency of insurance services for livestock and rangeland and not covering damages when claiming on the insurance can be of the most important reasons for this finding. Considering the close and undeniable relationship between climatic factors and forage production in rangelands, insurance services for livestock and rangeland can reduce part of pastoralists’ worries about their vulnerability to the effects of droughts^60,61^. Rangeland insurance in Iran faces challenges, such as inefficiency, weakness in damage assessment, and damages payment, causing most of the pastoralists to not have incentives for insurance services for livestock and rangeland, despite knowing about their vulnerability to climate change and its risks^62^. Therefore, accurate estimation of rangeland production in different climatic conditions can provide a basis for the development of the insurance industry, insurance pricing, and payment of damages. So, the criterion for payment of damages will be the average forage production of rangelands and its deviation from the effective rainfall index. In such conditions, a more suitable opportunity is provided for the organizations responsible for rangeland management in the climate crises to provide the necessary support for the development of the insurance industry in the livestock and rangeland sector. Therefore, it is necessary to prepare and formulate clear guidelines for livestock and rangeland insurance, so that rangeland or livestock damage will be paid for a claim in times of crisis.

The findings showed that the economic services and supports of the government had a significant effect on pastoralists’ vulnerability to climate change. This finding is in agreement with the results of other studies^39^. Therefore, the government’s lack of support for the pastoralists has a double importance in their low adaptive capacity to climate change^15,48,49^. Bad income status of pastoralists due to livelihood dependence on rangelands is the reason why they do not adopt well climate change adaptation strategies. The government can create alternative or complementary jobs for pastoralists to earn more money. In this regard, the appropriate allocation of government credits in the activities related to climate change in the rangeland sector plays an important role in the adaptation of the pastoralists. On the other hand, a lack of proper oversight of providing fodder to pastoralists in the climate crisis is one of the factors of their failure in adopting climate change adaptation strategies. The government is responsible for providing free fodder and medical and veterinary services to pastoralists which would require a subsidy^42,43,50,51,54^. In addition, the government can reduce the vulnerability of the water sector to climate change through providing water infrastructures (such as water pools and rainwater harvesting systems).

### The relationship between pastoralists’ personal characteristics and their weaknesses in adopting climate change adaptation strategies

The findings showed that with increasing age, the pastoralists’ weaknesses in adopting climate change adaptation strategies decreased. This finding is consistent with the results of other studies^21,50^. As age increases, pastoralists show more adaptive capacity in the face of climate change due to the indigenous knowledge acquired in different dimensions. In other words, taking advantage of indigenous knowledge may help pastoralists in adopting adaptation strategies and reducing their vulnerability to climate change^46,47,63^. Therefore, pastoralists have gained different experiences in facing the risks of climate change, especially drought, through various trials and errors, reducing their weaknesses in adopting climate change adaptation strategies^15^. Also, pastoralists who have a lot of experience in the face of climate change, have a higher understanding of adaptation strategies and natural hazards^39,50^.

The results showed that literate pastoralists had more adaptive capacity to climate change^22,41,53,54^. Less limitations of educated pastoralists in adopting climate change adaptation strategies compared to illiterate or less literate ones can be related to the ability of using education especially on cyberspace, and increasing knowledge and awareness in the use of various ways of adapting to climate change. Meanwhile, knowing the right time to use climate change adaptation strategies and timely response to natural hazards are the basis for reducing the weaknesses in adopting climate change adaptation strategies^3,44,52^. As the level of knowledge and access to climate information increase, pastoralists show a more principled attitude towards the resulting effects of climate change^21,50,55^. The high level of knowledge in the face of climate change will increase the rate of adaptation^3,38^. This requires providing targeted training to pastoralists and gaining their field experiences in the face of natural hazards^48,49^. Therefore, access to educational and extension services reduces pastoralists’ vulnerability to climate change^15,50,54^.

The pastoralists with more number of livestock had had less adaptive capacity to climate change than small pastoralists. Although a greater number of livestock can be a good source of income and savings in ideal climatic conditions, it faces pastoralists with serious challenges in environmental and climate crisis^15,21,53,54,57^.

The results showed that as income increases (from animal husbandry and non-animal husbandry), the pastoralists’ weaknesses in adopting climate change adaptation strategies decrease^15,21,41,53^. Therefore, strong financial and livelihood conditions plays an important role in pastoralists’ adaptation to climate change^48,49^. Having a secondary job will increase the amount of income and can help create employment for other family members^40,41^. Although, the lack of active labor is one of the challenges of the pastoralism^40–42^, it reduces the risks resulting from income and livelihood dependence on rangelands, which is a good way to adapt to climate change. Pastoralists owning agricultural equipment and tools (such as tractors) were more successful in overcoming some of the limitations of climatic fluctuations, especially water supply. This finding is consistent with the results of other studies^54^.

## Conclusion

Pastoralists’ livelihood is directly related to climatic variables in the rangeland. Climate change and its consequences such as droughts threaten pastoralism and have had negative impact on all dimensions of pastoralists’ livelihood. The low adaptive capacity of pastoralists in the face of climate change will worsen their livelihood status. Adopting climate change adaptation strategies can play an important role in the continuation of pastoralism and improving the sustainable livelihood of pastoralists. Therefore, it is of enormous importance to know the weaknesses of pastoralists in adopting climate change adaptation strategies. The adaptation of pastoralists in the face of climate change depends on external and internal weaknesses. Social and regulatory and insurance components were the most important internal and external weaknesses of the pastoralists’ failure in adopting climate change adaptation strategies, respectively. The external weaknesses played an important role in reducing the internal ones in the traditional pastoralism systems. Therefore, the adoption of new income activities other than animal husbandry and adjusting insurance services to match the pastoralists’ needs in the rangeland will increase pastoralists’ adaptive capacity in response to climate change. Determining the pastoralists’ livelihood capitals and their vulnerability to climate change could be ideas for future studies to improve adoption of climate change adaptation strategies by pastoralists.

## Data Availability

The datasets used and/or analyzed during the current study are available from the corresponding author on reasonable request.
